# Counting asbestos bodies in bronchoalveolar lavage: trend analysis and a systematic review

**DOI:** 10.1186/2049-3258-73-S1-P15

**Published:** 2015-09-17

**Authors:** Valerie Nuyts, Hadewijch Vanhooren, Kristiaan Nackaerts, Ben Nemery

**Affiliations:** 1 KU Leuven, Leuven, Belgium

## Background and aims

Asbestos bodies (AB) in bronchoalveolar lavage (BAL) can be quantified by light microscopy and their concentration is indicative of past cumulative asbestos exposure. The objective of this retrospective study was to assess clinical and exposure characteristics, as well as possible time trends, among patients in whom AB had been measured in BAL. We also conducted a systematic review of the literature on the subject.

## Methods

BAL samples were available from 548 subjects over a period from January 1997 until December 2013. The processing of samples and the microscopic analysis were done by a single expert and 75% of samples came from a single tertiary care hospital, allowing clinical and exposure data to be extracted from patient files. MEDLINE and Embase databases were searched for relevant articles on the subject, and 45 relevant articles were selected.

## Results

The study population (96% males) had a mean age of 62.2 (12.3) years. AB were detected in 56.4% of the samples, giving a median concentration of 0.5 AB.ml-1 (95th percentile: 25 AB.ml-1; highest value: 164.5 AB.ml-1). The AB concentration exceeded 1 AB.ml-1 in 40.1% and 5 AB.ml-1 in 18.6% of the samples. A significant decrease in AB concentrations was apparent over the years. High AB concentrations corresponded to high reported exposures to asbestos. AB concentrations were higher among patients with pleural disorders when compared to other disease groups. A systematic review of 45 published articles confirmed these observations: groups with occupational exposure to asbestos and patients with asbestos-related disease (ARD) had higher prevalences of positive BAL samples.

## Conclusions

This retrospective study of a large clinical population and a systematic literature review support the value of counting AB in BAL to assess past exposure to asbestos.

